# Operational Research to Assess the Real-Time Impact of COVID-19 on TB and HIV Services: The Experience and Response from Health Facilities in Harare, Zimbabwe

**DOI:** 10.3390/tropicalmed6020094

**Published:** 2021-05-31

**Authors:** Pruthu Thekkur, Kudakwashe C. Takarinda, Collins Timire, Charles Sandy, Tsitsi Apollo, Ajay M. V. Kumar, Srinath Satyanarayana, Hemant D. Shewade, Mohammed Khogali, Rony Zachariah, I. D. Rusen, Selma Dar Berger, Anthony D. Harries

**Affiliations:** 1 International Union Against Tuberculosis and Lung Disease (The Union), 75006 Paris, France; Pruthu.TK@theunion.org (P.T.); kctakarinda@gmail.com (K.C.T.); collins.timire@theunion.org (C.T.); akumar@theunion.org (A.M.V.K.); SSrinath@theunion.org (S.S.); HShewade@theunion.org (H.D.S.); sberger@theunion.org (S.D.B.); 2 The Union South-East Asia Office, New Delhi 110016, India; 3 AIDS and TB Department, Ministry of Health and Child Care, Causeway, Harare P.O. Box CY 1122, Zimbabwe; dr.c.sandy@gmail.com (C.S.); tsitsiapollo2@gmail.com (T.A.); 4 Yenepoya Medical College, Yenepoya, Mangalore 575018, India; 5 Special Programme for Research and Training in Tropical Disease (TDR), World Health Organization, 1211 Geneva, Switzerland; khogalim@who.int (M.K.); zachariahr@who.int (R.Z.); 6 Vital Strategies, New York, NY 10005, USA; irusen@vitalstrategies.org; 7 Department of Clinical Research, Faculty of Infectious and Tropical Diseases, London School of Hygiene and Tropical Medicine, London WC1E 7HT, UK

**Keywords:** COVID-19, Zimbabwe, Harare, presumptive tuberculosis, tuberculosis, TB treatment outcomes, HIV, antiretroviral therapy, EpiCollect5, operational research

## Abstract

When COVID-19 was declared a pandemic, there was concern that TB and HIV services in Zimbabwe would be severely affected. We set up real-time monthly surveillance of TB and HIV activities in 10 health facilities in Harare to capture trends in TB case detection, TB treatment outcomes and HIV testing and use these data to facilitate corrective action. Aggregate data were collected monthly during the COVID-19 period (March 2020–February 2021) using EpiCollect5 and compared with monthly data extracted for the pre-COVID-19 period (March 2019–February 2020). Monthly reports were sent to program directors. During the COVID-19 period, there was a decrease in persons with presumptive pulmonary TB (40.6%), in patients registered for TB treatment (33.7%) and in individuals tested for HIV (62.8%). The HIV testing decline improved in the second 6 months of the COVID-19 period. However, TB case finding deteriorated further, associated with expiry of diagnostic reagents. During the COVID-19 period, TB treatment success decreased from 80.9 to 69.3%, and referral of HIV-positive persons to antiretroviral therapy decreased from 95.7 to 91.7%. Declining trends in TB and HIV case detection and TB treatment outcomes were not fully redressed despite real-time monthly surveillance. More support is needed to transform this useful information into action.

## 1. Introduction

In early January 2020, a new coronavirus, named severe acute respiratory syndrome coronavirus 2 (SARS-CoV-2), was linked to a series of atypical pneumonia cases reported the previous month in Wuhan city, Hubei province, China. The virus spread rapidly within China, and then onto Europe, the United States of America (USA) and the rest of the world, causing the disease known as COVID-19. On 11 March 2020, the World Health Organization (WHO) declared COVID-19 to be a global pandemic. By the end of 2020, 80 million confirmed cases and 1.8 million deaths had been reported globally to the WHO, making COVID-19 the leading cause of death among infectious diseases [1]. In April 2020, one month after the pandemic had been declared, the epicenters were China, Europe and the USA, and with large volumes of air traffic between these regions and Africa, sub-Saharan Africa was predicted to be the next region to be hard hit by COVID-19 [2,3].

At the start of the COVID-19 crisis, political attention, resources and finances were redirected within the health sector to help it grapple with the escalating numbers of COVID-19 cases. National and local lockdowns restricted movement and forced people to spend more time indoors, limiting access to health facilities. All of this raised concern that countries with high burdens of tuberculosis (TB) and human immunodeficiency virus/acquired immune deficiency syndrome (HIV/AIDS) might see interruption of health services and poor quality of care for their patients [4]. Modeling studies suggested that deaths due to HIV/AIDS and TB could increase by up to 10 and 20%, respectively, with the greatest impact on HIV resulting from interruption to antiretroviral therapy (ART) and the greatest impact on TB resulting from delayed diagnosis and treatment of new cases [5].

Similarities were drawn with the Ebola virus disease outbreak in Sierra Leone and Liberia in 2014. Through restrictions in travel, zonal quarantines and understandable community fear of health facilities, the ability of the national TB programs to diagnose TB and continue with HIV testing of their TB patients was adversely affected, and, in the case of Liberia, there was a decline in the rates of TB treatment success [6,7]. HIV testing in the general population and in health facilities decreased in both countries, although access to ART remained unaffected [8,9]. Early on in the COVID-19 pandemic, the Stop TB Partnership and WHO issued guidance about how people with TB could protect themselves and how national TB programs might adjust to COVID-19 outbreaks and national/local lockdowns [10,11]. The United Nations Joint Programme on HIV/AIDS (UNAIDS) provided similar advice and guidance to people living with HIV [12]. This global advice was augmented by urgent calls for practical planning to tackle the growing threat of COVID-19 in sub-Saharan Africa [13].

The first COVID-19 case reported to WHO by Zimbabwe was on 21 March 2020. By 15 April, Zimbabwe had reported 18 COVID-19 cases (with three deaths) [14], and the country had gone into national lockdown. The impact that this would have on public health services for the management of TB and HIV/AIDS was unknown. The Zimbabwe National TB Programme (NTP) and National HIV/AIDS Programme (NAP) began preparations for the continued delivery of TB-HIV services in this new environment of restricted movement [15]. The country also drew on the example of Guinea in West Africa, which garnered resources to weather the Ebola virus disease storm and managed to maintain TB services despite numerous obstacles [16].

The Zimbabwe NTP and NAP, working in close collaboration with the International Union Against Tuberculosis and Lung Disease (The Union), the Special Programme for Research and Training in Tropical Disease at WHO (TDR) and Vital Strategies, decided to strengthen the routine and real-time monitoring and evaluation system for TB and HIV case detection. The quarterly (3-monthly) recording and reporting system was supported in selected health facilities in the capital city of Harare by recording and reporting every month. It was hypothesized that if there were declines seen in persons presenting with presumptive TB or in numbers registered and treated for TB, or decreases in persons presenting for HIV testing or in numbers of HIV-positive persons being referred for ART, then programs could act more quickly on monthly information rather than quarterly information to reverse these trends.

The overall aim of the study was to determine the impact of the COVID-19 pandemic on TB case detection, diagnosis and treatment outcomes and on HIV testing and referral to ART through strengthened real-time surveillance. In selected health facilities in Harare, Zimbabwe, specific objectives were on a monthly basis to: (i) document the cumulative monthly increase nationally in COVID-19 cases and deaths and the effects on general health services; (ii) collect, collate and report on specific TB and HIV-related data during the COVID-19 period (March 2020 to February 2021), (iii) document any specific programmatic responses at the national and local level to TB and HIV diagnosis and treatment during the COVID-19 period and (iv) compare the findings during the COVID-19 period with data collected and collated retrospectively during the pre-COVID-19 period (March 2019 to February 2020).

## 2. Materials and Methods

### 2.1. Study Design

This was a cohort study using programmatically collected aggregate data.

### 2.2. Setting

#### 2.2.1. General Setting: Zimbabwe and Harare

Zimbabwe is a low-income country in southern Africa with an estimated population of 14.6 million in 2019 and a gross national income per capita of USD 1390 [17,18]. About 70% of the population live below the poverty line. Zimbabwe is among the top 14 countries globally with a triple burden of TB, HIV and multidrug-resistant TB [19].

Harare is the capital city with an estimated population of 1.5 million [20]. The current study took place in Harare for two main reasons. The majority of cases of COVID-19 came from this area at the onset of the pandemic, and travel around the country was extremely difficult due to quarantine and prohibited travel outside of the city during the initial lockdown. Harare city consists of 8 districts, that include 46 public health facilities, of which 44 are under the Harare City Health Department. These provide general health services integrated with TB and HIV services whilst the other two are government central hospitals which provide specialized health services. Of the 44 public health facilities under the Harare City Health Department, 13 are polyclinics which additionally provide maternity services, 29 are satellite clinics and 2 are infectious disease hospitals (of which one became reserved as the city’s COVID-19 isolation center). Through stratified non-proportional sampling from each district, ten health facilities (nine polyclinics and one satellite clinic) were selected from a list of high-volume health facilities based on more than 1000 patients receiving life-long ART as a proxy for the volume of presumptive TB patients seen per year. The established staff who were already working in these sites delivering general health services and TB and HIV services assisted with the monthly collection of data.

#### 2.2.2. TB and HIV Services

The diagnosis and treatment of TB and HIV/AIDS in Zimbabwe are the responsibility of the NTP and the NAP, respectively, under the Ministry of Health and Child Care (MOHCC). People with symptoms suggestive of TB (typically, cough, fever, weight loss and night sweats) are classified as having presumptive TB when they attend a health facility. They are recorded as such in the presumptive TB register along with their demographic details. In the Harare city clinics, sputum samples are collected and sent to an onsite laboratory at each polyclinic, except satellite clinics that submit specimens to a laboratory at the nearest polyclinic. In the laboratories, patient details and sputum results are entered in the laboratory register. Investigations are carried out according to national and international guidelines [21,22], using the Xpert MTB/RIF assay (Cepheid, Sunnyvale, CA, USA) and/or sputum smear microscopy to establish a bacteriologically confirmed diagnosis of pulmonary TB (PTB), and results are sent back for patient tracking.

Those patients with a negative sputum result but who still have TB-related symptoms are referred to see a doctor at either of the two nearest infectious disease hospitals where clinical assessments, radiography and other circumstantial evidence are used to establish a diagnosis of clinically diagnosed PTB or extrapulmonary TB (EPTB). These patients are started on TB treatment before referral back to their initial health facility for registration and continuation of TB treatment. Patients with diagnosed TB are registered in the health facility Directly Observed Treatment (DOT) register and started on anti-TB treatment in accordance with national and international guidelines [21,22]. In the selected health facilities in Harare, patients with drug-susceptible TB (DS-TB) are treated and monitored with the standard 6-month regimens, while those with the drug-resistant disease are treated and monitored with MDR-TB regimens. Patients with drug-resistant TB were not included in this study. Treatment outcomes are monitored, recorded and reported according to international guidelines [23].

HIV testing takes place in public health facilities and is routinely offered to anyone attending for care, including TB patients, according to national and international guidelines [24,25,26]. HIV testing is carried out using rapid testing algorithms in line with WHO guidance, and test results are entered in the HIV Testing Services (HTS) register that is placed at all health service delivery points. Once patients are diagnosed positive for HIV, they are retested for verification of HIV diagnosis and, if confirmed HIV-positive, they are then prepared and counseled for ART and referred for immediate start of ART regardless of WHO clinical stage or CD4 cell count. Administration of an HIV screening tool for HIV risk assessment before providing a rapid HIV test and use of HIV self-testing are measures that have been scaled up recently to improve the HIV positivity yield.

There is generally good quality data capture and reporting for TB and HIV/AIDS at all levels due to regular supervision by national program supervisors.

#### 2.2.3. Data Monitoring, Recording and Reporting for the Study in Health Facilities in Harare

Data were routinely collected daily by front-line health workers. They provided services in each of the ten health facilities in Harare using the standard MOHCC monitoring tools (presumptive TB register, sputum laboratory register, TB patient register and HTS register), most of which were paper-based. One to two weeks after the end of each month, the project country coordinator (KCT—who was appointed specifically for the study) visited each site along with his team of trained data collectors. They collated the individual data on TB and HIV variables for the previous month into monthly aggregate data, which they then entered into a proforma developed using an EpiCollect5 application (https://five.epicollect.net, accessed on 29 May 2021).

For TB treatment outcomes, the monthly cohorts of patients enrolled onto treatment eight months previously were used—this allowed for six months of treatment to be completed and a further two months for outcomes to be validated and recorded in the registers. For example, the October 2020 TB treatment outcome data were obtained for TB patients enrolled and started on treatment in February 2020. This allowed for clear separation of treatment cohorts in the pre-COVID-19 and COVID-19 periods.

National data on COVID-19 cases and deaths reported to WHO on the last day of the month were obtained from WHO situation and epidemiological reports [1].

When the prospective monthly data were collected, a schedule and the same procedures were used to collect retrospective data. For each month of the COVID-19 period (March 2020 to February 2021), data were collected on TB and HIV parameters for a matching period one year before the COVID-19 period (March 2019 to February 2020).

Once all prospective and retrospective data for the month had been entered into EpiCollect5, they were checked and validated by both the project country coordinator and the overall project monitoring and evaluation officer (PT) based at The Union. Data were then presented in a monthly report as a series of figures and tables and narrative to the directors of the NTP and NAP and to all other relevant stakeholders involved in the project. Key policy or practice changes made at the local health facility or at the national level during that month to explain and/or counteract the effects of COVID-19 on TB and HIV parameters were recorded in a narrative table within the report. These monthly reports were always sent and received by the national program staff within four weeks of closure of that month to enable timely surveillance and possible action.

### 2.3. Study Population

The study population included all patients presenting to TB services with presumptive TB, all TB patients registered for DS-TB treatment and all persons tested for HIV between March 2019 and February 2021; March 2020 to February 2021 was the COVID-19 period, and March 2019 to February 2020 was the pre-COVID-19 period. We also included TB patients registered for treatment eight months prior to the study period to assess treatment outcomes.

### 2.4. Data Variables, Sources of Data and Timing of Data Collection

Data variables for TB included aggregate numbers of patients: with presumptive PTB, stratified by male and female, adults (≥15 years) and children (<15 years); who were diagnosed with bacteriologically positive PTB by either smear microscopy and/or Xpert MTB/RIF; with registered TB, stratified by bacteriologically confirmed PTB, clinically diagnosed PTB and EPTB; and with registered TB, who were newly tested for HIV in that month after being diagnosed with TB—this excluded patients who already knew they were HIV-positive or had recently been diagnosed HIV-negative. Standardized TB treatment outcomes of those patients enrolled for treatment eight months previously were collected and included—treatment success (a combination of those cured and those who completed treatment with no sputum smear examination), lost to follow-up (LTFU), died, failed treatment or not evaluated [23]. LTFU refers to a TB patient who did not start treatment or whose treatment was interrupted for two consecutive months or more. Not evaluated is an outcome given to those who transfer from one facility to another and for whom the final treatment outcome is not recorded and also to those whose outcome is not recorded and unknown to the reporting unit.

Data variables for HIV included: persons who were HIV tested at the health facilities, stratified by male and female, adults (≥15 years) and children (<15 years); persons diagnosed HIV-positive; and HIV-positive persons referred to ART services.

Sources of data were the presumptive TB register, the sputum laboratory register, the TB patient register, and the HTS register. Prospective and retrospective data for the study were collected between June 2020 and March 2021.

### 2.5. Analysis and Statistics

Aggregate data were entered in EpiCollect5, where they were checked and validated in-country by the country coordinator and by The Union’s monitoring and evaluation officer. Data were presented as frequencies and proportions, and comparisons were made between the COVID-19 period and the pre-COVID-19 period. The percentage decline in numbers during each month of the COVID-19 period was calculated relative to the numbers during the same month of the pre-COVID-19 period. The relative percentage differences observed between the first 6 months of COVID-19 (March to August 2020) and the second 6 months of COVID-19 (September 2020 to February 2021) were also calculated.

## 3. Results

### 3.1. COVID-19 Cases and Deaths and General Effects on Health Services

**Figure 1:**
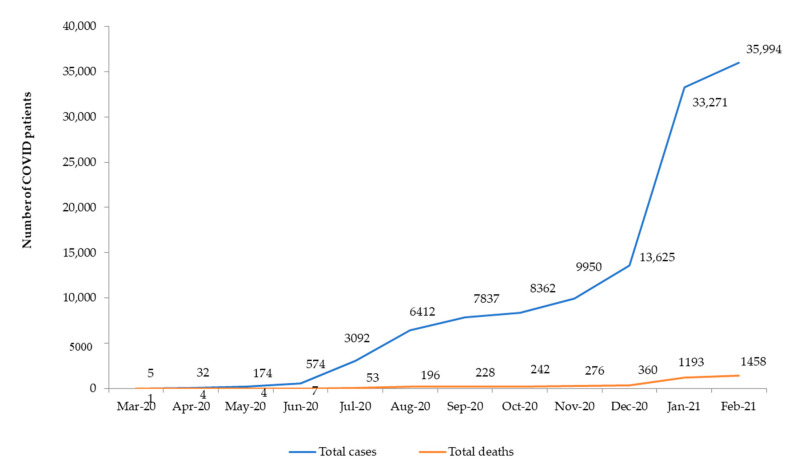
Cumulative number of COVID-19 cases and deaths in Zimbabwe between March 2020 and February 2021 as reported to the World Health Organization.

COVID-19 cases and deaths as reported to WHO cumulatively increased to 35,994 and 1458, respectively, during the 12 months (see Figure 1).

*From a general services perspective*, there had been heightened economic decline in Zimbabwe in the pre-COVID-19 period, especially towards the end of 2019 and start of 2020. This resulted in a series of health care worker strikes which affected service provision. The introduction of flexible working hours in 2020 helped to restore health service delivery. In March 2020, COVID-19 struck. There was a national lockdown between March and the end of June 2020 and again from 6 January to 28 February 2021. During lockdowns, people were not allowed to leave their places of residence, public transport ceased, there were night curfews, health facilities shortened their working hours, and some health facilities had to close due to either shortage of health care workers or if there was an outbreak of COVID-19 within the facility. Only a few patients were allowed into health facilities at a time, resulting in long waiting queues outside, which may have deterred patients from coming to the health facilities. Between July and September 2020, there was industrial strike action again in the health sector over the lack of personal protective equipment and remuneration. Locum staff brought in to fill the gaps were not familiar with programmatic activities, including recording and reporting. There was widespread community fear about contracting COVID-19 or being diagnosed with COVID-19 at health facilities during the whole period.

*From a TB perspective*, environmental health technicians, responsible for community follow-up of TB patients while on treatment, were repurposed to COVID-19 activities. Between 12 December 2020 and 11 January 2021, GeneXpert cartridges expired in some facilities, and during this time clinic staff did not collect sputum specimens for TB investigation.

*From an HIV perspective*, voluntary male medical circumcision services (VMMC), which provided HIV testing for a large number of male adolescents and men, stopped in March 2020 and remained closed for the next 12 months. There were stock-outs of HIV test kits in July 2020, which remained a challenge intermittently for the next few months.

### 3.2. TB Case Finding, Diagnosis and Registration

There was an overall decrease in the numbers of persons presenting with presumptive PTB (40.6%), people diagnosed with bacteriologically confirmed PTB (30.1%) and people being registered for TB treatment (33.7%) in the COVID-19 period compared to the pre-COVID-19 period (Table 1).

For presumptive PTB, the overall decrease was greater in children (71.3%) compared with adults (38.6%), but almost similar between males (38.4%) and females (36.0%). The yield of bacteriologically positive PTB in those investigated for presumptive TB increased from 13.5 to 15.9%. The decline in those diagnosed and registered with TB was worse for patients with PTB, and amongst those with pulmonary disease, it was worse for those with clinically diagnosed PTB (46.0%). The proportion of TB patients tested for HIV remained above 90%, although it declined from 95.1 to 90.3%.

**Figure 2:**
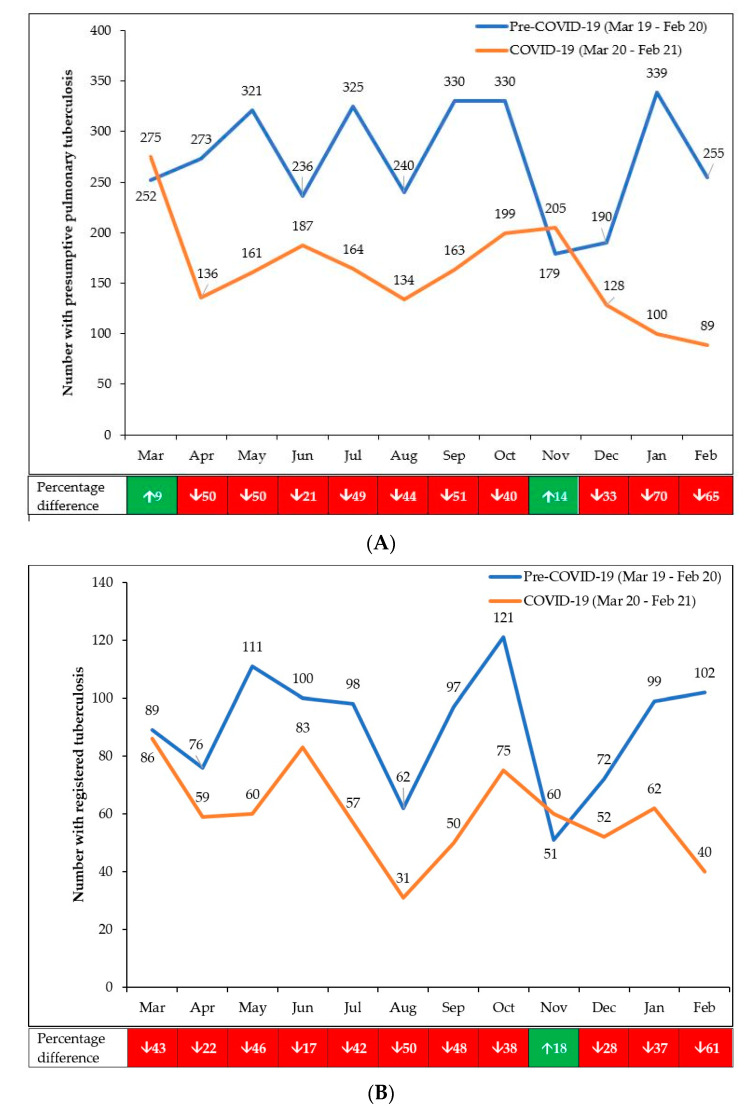
(**A**): Monthly numbers of persons presenting with presumptive PTB in 10 health facilities in Harare, Zimbabwe, during pre-COVID-19 and COVID-19 periods. (**B**): Monthly numbers of persons presenting with registered TB in 10 health facilities in Harare, Zimbabwe, during pre-COVID-19 and COVID-19 periods. ↑ = increase; ↓ = decrease. Interventions applied from July 2020 onwards to counteract the decline in numbers included: integrated screening of patients with respiratory symptoms for TB and COVID-19; improved contact tracing of index patients with TB and COVID-19; and strict infection control practices at health facilities which were promoted to try and encourage symptomatic patients to attend.

The monthly numbers of persons presenting with presumptive PTB and registered TB in the pre-COVID-19 and COVID-19 periods are shown in Figure 2A,B. The footnotes for the Figures indicate the interventions that were put in place to counteract the downward trends.

Compared with the pre-COVID-19 period, the decline in presumptive TB in the first 6 months of COVID-19 (March to August 2020) was 35.8%, and this became greater in the second 6 months (September 2020 to February 2021) when the decline was 45.5%. The decline in registered TB in the first 6 months of COVID-19 was 29.9%, which also became greater in the second 6 months when the decline was 37.5%.

### 3.3. TB Treatment Outcomes

The overall aggregate treatment outcomes between the pre-COVID-19 and COVID-19 periods are shown in Table 2. There was a decrease in treatment success from 80.9 to 69.3%, mainly due to an increase in patients “not evaluated” (12.1%). Other program outcomes were similar between the two periods.

**Figure 3:**
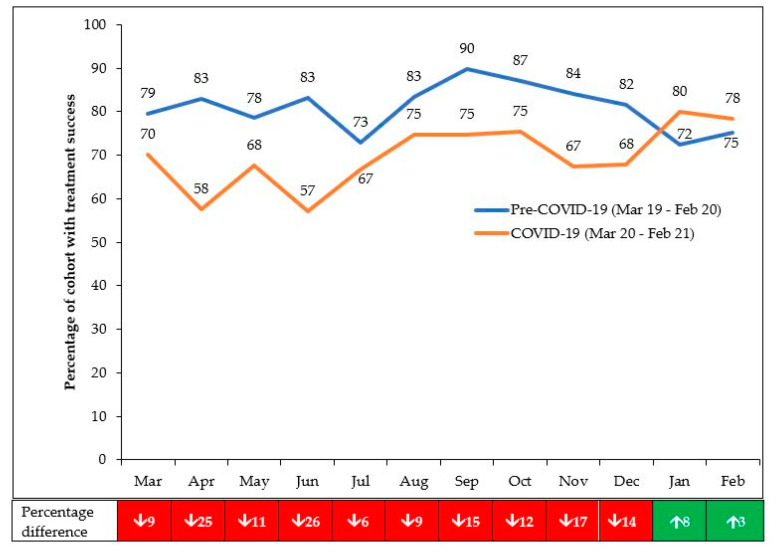
Treatment success amongst patients enrolled each month in 10 health facilities in Harare, Zimbabwe, during pre-COVID-19 and COVID-19 periods. Enrollment occurred eight months prior to the month of reporting (to allow for 6 months treatment and 2 months of follow-up and record the final outcome); ↑ = increase; ↓ = decrease. The interventions applied from July onwards included: medication refills given for longer periods and synchronized for TB and ART medications; attempts to reduce the outcome “not evaluated”.

The monthly treatment success rates in the pre-COVID-19 and COVID-19 periods are shown in Figure 3. Interventions to reverse downward trends are indicated in the footnotes. Compared with the pre-COVID-19 period, the decline in treatment success was 14% in the first 6 months of COVID-19. This improved in the second 6 months when the decline was 7.8%. Treatment success particularly improved in January and February 2021 to 80 and 78%, respectively.

### 3.4. HIV Testing and Referral to ART

There was a large overall decrease in numbers tested for HIV between pre-COVID-19 and COVID-19 periods (Table 3). The overall decrease was greater for children (70.2%) compared with adults (62.4%) and greater for males (79.1%) compared with females (53.6%). There was a small relative increase in the HIV positivity rate from 6.0 to 8.1% and a small decrease in the referral of HIV-positive persons to ART from 95.7 to 91.7% during the COVID-19 period.

**Figure 4:**
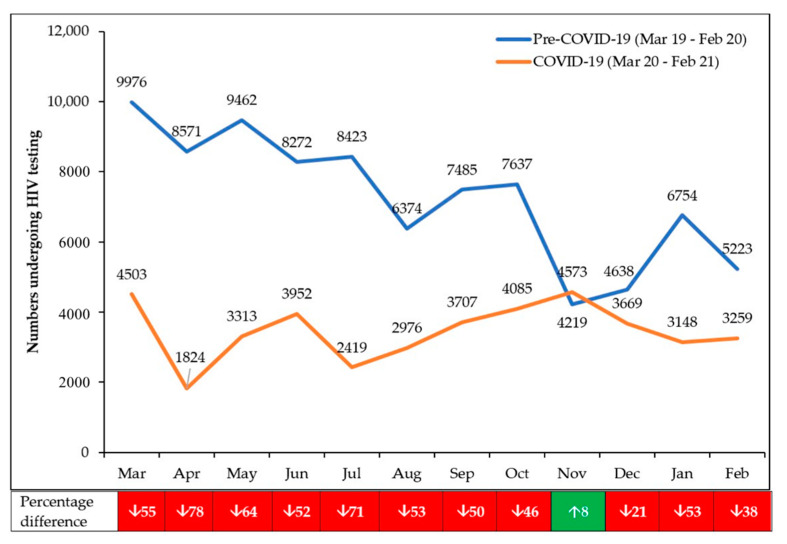
Monthly numbers presenting each month for HIV testing in 10 health facilities in Harare, Zimbabwe, during pre-COVID-19 and COVID-19 periods; ↑ = increase; ↓ = decrease. From August 2020 onwards, human resources support was given to the Ministry of Health by PEPFAR partners (President’s Emergency Fund for AIDS Relief in Africa) by seconding direct service delivery nurses to affected sites.

The monthly numbers tested for HIV in the pre-COVID-19 and COVID-19 periods are shown in Figure 4.

The number of people being HIV tested in health facilities was already declining in the pre-COVID-19 period due to a number of factors that included: a shift from general HIV testing to more targeted HIV testing using a screening tool to identify high-risk groups, to identify those with a high lifestyle risk score assessment and to identify those who had not been tested in the previous year; promotion of HIV self-testing, with the referral of only those HIV-positive to the health facilities for confirmation of the result; and a greater focus on index partner testing. As explained in the footnotes of Figure 4, human resources support was provided to clinics from August 2020 onwards to sustain HIV testing services and ART delivery. Compared with the pre-COVID-19 period, the decline in HIV testing in the first 6 months of COVID-19 was 62.8%, which was greater than the 37.6% decline observed in the second 6 months.

## 4. Discussion

This is the first study in Zimbabwe to assess the impact of COVID-19 on TB and HIV services in selected health facilities in Harare, the capital city. In summary, despite attempts by the NTP, the NAP and other parts of the health sector to prepare for COVID-19 [15,27], there was a large negative impact on almost all aspects of TB case detection, diagnosis and treatment, as well as HIV testing. The referral of HIV-positive persons to ART was also affected but only to a small degree. A health service already under strain was hit hard and brought almost to its knees in the first 12 months of the COVID-19 pandemic [27].

With respect to TB program activities, the numbers of people presenting to health facilities with presumptive PTB declined considerably over the 12-month COVID-19 period. These findings were similar to those described in the early months of COVID-19 in clinics in Tehran, Iran [28] and Nigeria [29], where lockdown restrictions, transportation difficulties and community fear of health facilities were thought to hinder health facility access. There was a corresponding decline in numbers diagnosed and registered with TB, in line with reports from other countries where the decreases in TB case notifications in the early months of COVID-19 compared with previous years were 48% in clinics in China [30], 48% in clinics in Brazil [31] and 56% in India [32].

A modeling analysis at the start of the pandemic suggested that a 3-month suspension of TB services due to COVID-19 lockdown followed by ten months restoration back to normal would cause over five years an additional 1.2 million TB cases in India, 25,000 additional TB cases in Kenya and 4000 additional cases in Ukraine, mainly as a result of the accumulation of undetected TB during lockdown [33]. Unfortunately, in Zimbabwe, there was no restoration of services back to normal. On the contrary, from October 2020 to February 2021, there was a steady decline in numbers of persons with presumptive PTB and registered TB, coinciding with the expiry of cartridges for Xpert MTB/RIF assays. Sputum specimens were not collected for investigation, and patients with TB-related respiratory symptoms stayed away. The one encouraging finding was an overall increase in bacteriological positivity in the COVID-19 period compared with the previous year. There are several possible explanations that include: (i) when laboratories were functioning and reagents were available, standard laboratory operating procedures remained intact; (ii) there was selective self-referral of only those who were really sick with TB; and (iii) patients may not have had access to community TB testing services as a result of COVID-19, and therefore those with TB came to Harare city health facilities for investigation.

TB treatment success rates declined during the COVID-19 pandemic. However, there was some improvement in the second half of the year, and particularly in January and February 2021, when outcomes were better than at the corresponding times before COVID-19. The probable reason for better outcomes at this time was, as discussed earlier, a series of health care worker strikes towards the end of the pre-COVID-19 period as a result of heightened economic decline. This disrupted routine follow-up of TB patients on treatment, resulting in a high number of patients not being evaluated.

With the onset of the COVID-19 pandemic, we were concerned that COVID-19 restrictions would hinder patients collecting anti-TB medications and compromise drug adherence. However, the longer periods given between anti-TB drug refills and their synchronization with ART medication refills made things easier for patients and partly mitigated this challenge. The large proportion of patients “not evaluated” was a huge challenge, compromising the program’s ability to record successful treatment outcomes. This was partly due to the environmental health technicians responsible for following up patients in the community being repurposed to COVID-19 activities. Concerted efforts, however, were made to tackle this issue, and through human resources support from PEPFAR (the President’s Emergency Plan for AIDS Relief), treatment success rates considerably improved in the last two months.

With respect to HIV services, there was a significant decrease in numbers of people presenting for HIV testing, although this did improve during the latter half of the COVID-19 period. There were two main reasons for the large decline. First, the cessation of VMMC services would have resulted in a large decline in males being HIV tested during this time. Second, in April 2020, during the national lockdown, the MOHCC issued a directive for all community-based HIV testing to be temporarily halted to minimize the risk of community health workers and community members from contracting COVID-19. The improvement in the latter half of the COVID-19 period may have been due to the strong support from PEPFAR partners.

The overall reductions in HIV testing were similar to what has been observed in Europe [34], the USA [35] and Africa [36]. It is likely, though, that we have overestimated the COVID-19 impact on HIV testing because of Zimbabwe adopting a more targeted approach, promoting HIV self-testing and focusing on index HIV testing before the onslaught of COVID-19. The increase in HIV positivity observed in the COVID-19 period in our study is probably a result of this more targeted approach to identifying clients more at risk of HIV. While referrals to ART decreased in the COVID-19 period, it was encouraging to see that they were still maintained at over 90%.

There are several strengths to this study. First, the real-time monthly surveillance was embedded within the routine services of the health facilities, and there was cross-checking and validation of the data each month between the country coordinator and the overall study monitoring and evaluation officer. We believe, therefore, that the data were accurate and reflected programmatic practice in the field in Harare. Second, we used two 12-month periods to compare data, thus accounting for seasonal changes that might have affected TB case detection and HIV testing. Third, the conduct and reporting of the study were in line with the Strengthening the Reporting of Observational Studies in Epidemiology (STROBE) guidelines [37].

However, there were some limitations. Our study was limited to health facilities in Harare, and therefore may not be representative of Zimbabwe as a whole. The use of aggregate quantitative data made it impossible to understand the cascade of care for TB case detection and HIV testing during COVID-19. Further mixed-methods research with both quantitative and qualitative analysis amongst TB patients themselves, as has been conducted in Zambia [38], are needed to obtain a better idea of why patient access to health services and health service delivery was so badly affected. We only assessed referrals of HIV-positive persons to ART and did not document the initiation or retention on ART. Previous studies have suggested that ART interruption has been a problem during the COVID-19 pandemic [39]. It would have been interesting to assess this in Zimbabwe, especially as interruption to ART is thought to be one of the most important determinants of HIV-related mortality during the COVID-19 pandemic [40]. Finally, the official monthly reports to WHO of COVID-19 cases and deaths may have underestimated the true burden of COVID-19 in the country. A study on deceased people at the University Teaching Hospital morgue in Lusaka, Zambia, found that just 9% of 70 people, who were confirmed as having SARS-CoV-2 from postmortem nasopharyngeal swabs within 48 h of death, had ever been tested before death [41]. This finding is unlikely to be confined to Zambia, and large numbers of unreported cases and deaths due to COVID-19 are probably occurring in other countries in Africa.

Despite these limitations, there are some important programmatic implications from this study. First, the strengthened monthly surveillance system worked well with both NTP and HIV program directors looking each month at the monthly reports and using the data for decision making and suggesting interventions. The monthly data were also shared with the ten health facilities through the country coordinator, and this enabled the health facilities to implement interventions simultaneously. However, the monthly surveillance and the interventions applied were insufficient to reverse the direct negative effects of COVID-19 on services or the indirect effects such as industrial action and stock-outs of diagnostic tests. Given the challenges both programs faced, it is remarkable that services were able to function at all, so without monthly surveillance, it might have been much worse. The monthly surveillance as conducted in Harare required effort and external support. If this became routine as desired, the NTP would require considerable investment, probably involving electronic case-based reporting systems. Technical support by partners, who have additional human resources to help, would be vital to get activities and services back to normal.

Second, with COVID-19 likely to become endemic, there is an urgent need to bring TB case detection and treatment outcomes back to pre-COVID-19 levels. This study suggested that better contact tracing of TB patients and heightened attention to reducing the “not evaluated” category were important interventions for TB case detection and TB treatment outcomes. Support from PEPFAR partners also helped to support staff in ascertaining TB treatment outcomes. There are several other suggestions about how TB services could be sustained or improved in the face of the pandemic. These include: better integration and screening of TB and COVID-19 at the health facility and community level; sharing testing algorithms and multiplexing equipment such as GeneXpert platforms within the laboratories; having robust procurement and delivery systems to avoid reagent stock-outs; ensuring effective infection, prevention and control activities within health facilities; having longer 3-month follow-up appointments for patient check-ups and drug collection; providing health information education in health facilities and the community; and mobilizing support networks of TB survivors and TB communities [42,43,44]. More use of digital platforms for case finding, drug adherence, management of adverse drug reactions and training have also been recommended as a way of rapidly restoring TB diagnosis, care and prevention services [45]. The Zimbabwe NTP and MOHCC could consider which, if any, of these innovative approaches might help, and might be feasible and cost-effective to implement.

Third, HIV self-testing, index partner notification and home-based HIV testing services have allowed HIV testing numbers in the COVID-19 era to rebound in some African countries [46,47,48]. Zimbabwe is already moving in this direction. It is also vital to capture all HIV testing and HIV test results outside of as well as within the health sector in order to keep informed about what is happening and ensure that all HIV-infected persons are able to initiate ART. Zimbabwe again is already implementing such an approach and is tracking on a monthly basis facility and community distribution of HIV test kits, as well as numbers tested for HIV along with their results. Further deployment of electronic data capture systems will be important in this context.

Finally, the resources and funding that have been raised to fight the COVID-19 pandemic have been staggering. The proposed global financing systems that have been proposed as a way of promoting and enabling science and product development in the health sector [49] must be used and leveraged to help fight other diseases, TB and HIV/AIDS included, so that we can stay on track to deliver on the Sustainable Development Goals in 2030.

## 5. Conclusions

Using strengthened monthly real-time surveillance in 10 health facilities in Harare, Zimbabwe, we documented that numbers of persons with presumptive PTB and registered TB, treatment outcomes of patients enrolled on treatment and numbers being HIV tested all declined during 12 months of the COVID-19 outbreak compared with 12 months pre-COVID-19. The referral of HIV-positive persons to ART also declined, although they were maintained at high levels throughout. Despite using the monthly data, the declining trends in TB and HIV services could not be reversed because of on-going restrictions resulting from the COVID-19 pandemic combined with industrial action in the health sector and the expiry of certain diagnostic reagents. Suggestions have been made as to how to restore TB case detection and HIV testing so that TB- and HIV-related morbidity and mortality can be kept as low as possible during this difficult period.

## Figures and Tables

**Table 1:** Characteristics of persons with presumptive pulmonary TB and registered TB in 10 health facilities in Harare, Zimbabwe, during pre-COVID-19 and COVID-19 periods.

Numbers of Patients	Pre-COVID-19 Mar 2019–Feb 2020 N	COVID-19 Mar 2020–Feb 2021 N	Difference between Pre-COVID-19 and COVID-19 %
Presumptive pulmonary TB:	3270	1941	↓40.6
Adults (≥15 years)	3013	1849	↓38.6
Children (<15 years)	181	52	↓71.3
Male	1866	1150	↓38.4
Female	1221	782	↓36.0
Bacteriologically positive	442	309	↓30.1
Positivity rate (%)	(13.5%)	(15.9%)	↑2.4% *
Registered TB:	1078	715	↓33.7
Bacteriologically confirmed PTB	528	346	↓34.5
Clinically diagnosed PTB	465	251	↓46.0
Extrapulmonary TB	136	97	↓28.7
Eligible for being newly HIV tested	586	423	↓27.8
Newly tested for HIV (%)	(95.1%)	(90.3%)	↓4.8% *

* absolute change (increase or decrease); TB = tuberculosis; PTB = pulmonary tuberculosis; ↑ = increase; ↓ = decrease.

**Table 2:** Treatment outcomes of patients enrolled in TB treatment in 10 health facilities in Harare, Zimbabwe, during pre-COVID-19 and COVID-19 periods.

Treatment Outcomes in Patients Enrolled for TB Treatment	Pre-COVID-19 Mar 2019–Feb 2020 n	COVID-19 Mar 2020–Feb 2021 n	Difference between Pre-COVID-19 and COVID-19 %
Enrolled for treatment:	1210	979	
Treatment success (%)	(80.9)	(69.3)	↓11.6 *
Lost to follow-up (%)	(2.0)	(2.3)	↑0.3 *
Died (%)	(4.3)	(3.7)	↓0.6 *
Failed (%)	(0.6)	(0.4)	↓0.2 *
Not evaluated (%)	(12.2)	(24.3)	↑12.1 *

* absolute change (increase or decrease); TB = tuberculosis; ↑ = increase; ↓ = decrease; treatment outcome was considered “treatment success” when the TB patient was either cured or had “treatment completed”. The success rate was calculated for the month-wise cohort of TB patients commenced on treatment eight months prior to the reporting month (this takes account of six months of treatment to be completed and another two months to finalize the recording of the final treatment outcome).

**Table 3:** Characteristics of persons tested for HIV and referred to antiretroviral therapy in 10 health facilities in Harare, Zimbabwe, during pre-COVID-19 and COVID-19 periods.

Characteristics	Pre-COVID-19 Mar 2019–Feb 2020 n	COVID-19 Mar 2020–Feb 2021 N	Difference between Pre-COVID-19 and COVID-19 %
Underwent HIV testing at a health facility:	51,078	18,987	↓62.8
Adults (≥15 years)	48,661	18,289	↓62.4
Children (<15 years)	2316	691	↓70.2
Male	18,278	3816	↓79.1
Female	32,706	15,171	↓53.6
Positive for HIV	3045	1547	↓36.7
HIV positivity rate (%)	(6.0%)	(8.1%)	↑2.1 *
Referred to ART (%)	(95.7%)	(91.7%)	↓4.0 *

* absolute change (increase or decrease); ↑ = increase; ↓ = decrease; ART = antiretroviral therapy.

## Data Availability

The data that support the findings of the study are available from one of the first authors (P.T.) upon reasonable request.
